# Editorial: Climate change and society

**DOI:** 10.3389/fsoc.2022.991193

**Published:** 2022-08-10

**Authors:** Fátima Alves, Luisa Schmidt

**Affiliations:** ^1^Universidade Aberta, Lisbon, Portugal; ^2^Centre for Functional Ecology and TERRA Associated Laboratory, University of Coimbra, Coimbra, Portugal; ^3^Institute of Social Sciences, University of Lisbon, Lisbon, Portugal

**Keywords:** climate change, social sciences, public perceptions, climate action, climate change policies, social inequalities

Climate change presents one of the greatest challenges of the 21st Century. It will massively affect human societies in complex and multiple ways. And it seems to be almost uncontrollable in the near future. Our knowledge of the chemistry and physics of climate change, its causes and its consequences for planetary systems, is far greater than our understanding of the societal changes it poses. Climate change results from a complex process of societal transformations, which we all need to understand to better cope with the challenges it presents. Climatic conditions play a significant role and interfere with people's lives in multiple ways. The causes are essentially known, based on unequivocal human action. All solutions also involve human decision and action. It is social and human action in both individual and social settings that are decisive for the future pathways of climate change and its disentanglement.

There is also a factor of climate injustice that must be addressed. The nations that contributed most to the problem are often those that experience more limited and manageable consequences while those who contributed the least are often the most affected, vulnerable and unprepared. With climate change, the risk of conflicts, disasters and internal displacement increases so exacerbate existing inequalities and poverty. This presents a moral conundrum of the highest order.

At the ecological level, the destruction or disintegration of nature/nurture is more visible, with strong impacts on the availability and reduction of natural resources. In terms of social systems, climate change breaks down social organization, housing, the food system, generates migration, increases economic losses, hunger and public health breakdowns. In a more invisible way, climate change destroys cultural belonging and individual and collective identities. In addition to these expected impacts in the most diverse social, cultural, economic and environmental sectors, human health has emerged as an important area of considerable alarm. Although not frequently mentioned or targeted as a key political concern, it is expected that the impact of climate change on human health will be severe, both in the proliferation and incidence of diseases. Moreover, climate change will have extensive implications to human wellbeing, which will reflect on social structures and ways of life.

Though the impact of changes in these biophysical systems is widely recognized, as it is increasingly in economies, at a social and cultural level and when most needed, there is still a long way to go. This situation adds to the complexity of the challenges of the climate emergency, insofar as it introduces contextual, territorial diversities and social-cultural diversities of the people who inhabit those territories, and with that, social inequalities.

For a long time, the importance of a paradigm shift has been widely recognized, in which societies face the paradox of continuing to deepen the well-documented socio-ecological crisis whilst it is imperative to alleviate it. We seek, but we do not find, leadership from the world's rulers. The need for transformative change is an imperative from which we can no longer escape. The alternative is catastrophe or action on top of it.

The aim of this Research Topic is to highlight and share knowledge on the social, economic, political and cultural implications of climate change, as well as reflect upon the required transformations in policy, governance and social-cultural strategies to accelerate mitigation, adaptation and prevention. To understand the multiple dimensions of climate change and their interdependencies, we need to bring together a multitude of sciences, knowledges, powers, and decision-makers.

The social sciences and sociology play a central role in analyzing the effects of human activities on natural systems. Social Sciences can scrutinize those phenomena and relations that, within human societies, produce social structures and ways of thinking and judging that ultimately undermine the integrity of the environment.

This Research Topic provides an overview of social sciences, and also multidisciplinary literature and research on climate change and socio-ecological challenges, facilitating the identification of key areas for further research and development. The articles included in this Research Topic address a variety of themes seeking to clarify the need to understand and act on climate change. They question forms of knowing and the understanding of the senses and meanings, perceptions, and the role of social and cultural factors in their construction. Thus, this Research Topic extends to the knowledge of perceptions, to the need to convey meaningful voice to the voiceless, whether children, women, or any other minorities, in order to highlight the processes of good governance and citizen participation, and to ensure supportive involvement in environmental decisions. These are prerequisites of the ecological transition requiring transformative changes at structures' and subjects' levels. The aforementioned Research Topic includes 9 articles (organized) as follows:

Kythreotis et al. analyse “*Citizen Social Science for More Integrative and Effective Climate Action: A Science-Policy Perspective*,” highlighting the challenges to States and policies to keep the temperature below 2°C, focusing on the role of citizens and citizen agency in changing behaviors. The authors seek to elevate “Citizen Social Science (CSS) to a new level across governments as an advanced collaborative approach to accelerating climate action and policies that moves beyond conventional citizen science and participatory approaches.” In this context, citizens become the central actors in driving climate policy change.

Romero-Lankao and Gnatz, link adaptation to climate change with SDG and the New Urban Agenda. The article “*Risk Inequality and the Food-Energy-Water (FEW) Nexus: A Study of 43 City Adaptation Plans*” suggests relating inequality in climate risk to urban populations. The authors “examine whether and how adaptation plans from C40 member cities address inequality in risk, by planning actions to reduce hazard exposure or tackling the drivers of social vulnerability.” In general, their findings express that the “FEW-nexus thinking is not yet embedded in narrative understandings of risk and planned adaptation actions, within the adaptation plans” they have studied.

In their essay, Aldeia and Alves discuss the limits of mainstream environmental sociology as a field capable of fostering how we understand and deal with contemporary socio-ecological problems. They argue how Western capitalist modernity is premised upon a fundamental separation of Society and Nature that transforms the latter into the mere environment of the Anthropos, something which transforms the environment into a resource pool for modern capitalist exploitation. Rejecting the idea that we are currently experiencing an environmental crisis, the authors reason that we are rather living through “a crisis of Western modernity itself and of the kind of worlds that are possible and impossible to build within it.” As such, the environment is not what needs to be saved but quite the opposite: it is a subject that actively needs to be un-thought if our current world(s)-building crisis is to be overcome. “*Against the Environment. Problems in Society/Nature Relations*” contributes to critically “unthink” both mainstream environmental sociology and the ways in which modern capitalist worlds are made. In doing so, this paper directs our attention to alternative possibilities for enacting multiple and interconnected ontologies of humans and non-human life.

In “*Perceptions of Local Environmental Issues and the Relevance of Climate Change in Nepal's Terai: Perspectives from Two Communities*,” Nash et al., investigates community perceptions and representations of environmental and climate-relevant issues that are critical to underpinning responses to climate change, within two communities in the Terai region of Nepal: Bharatpur and Kumroj in Chitwan Province, having conducted 30 qualitative interviews with local people. Results highlight that “climate change is yet to penetrate the environmental representations of some communities and there is a need to address the disconnect between local issues and global climate change.” The need to make climate change relevant locally, particularly for communities at risk, brings new directions for the development of action and a novel policy agenda.

Signoretta et al., in “*Fiddling While Rome Burns”: The Role of Ecological States in the Association Between Greenhouse Gas Emissions and Subjective Well-Being*,” analyze the hypothesis that the ecological state produces a positive association between greenhouse gas emissions and mental wellbeing. The authors examine this in the context of the countries of the European Union using a hierarchical three-level analysis on the third wave (2011–2012) of the European Quality of Life Survey for a sample of EU citizens.

The findings support their hypothesis, that individuals in all ecological states continue to treat climate change as an environmental and economic trade-off. In the end, the study calls for the emergence of action on climate change issues.

Murphy et al., in ““*That's Where Our Income Comes From”: Women's Perceptions of Links Between Reproductive Struggles and Hydraulic Fracturing*,” analyses how the causes and consequences of the environmental crisis experienced globally are at the root of climate change, but also of the reproductive difficulties that she takes as a case study in her article. The author departs from the great stigmatization around reproductive difficulties, usually relegated to the private sphere, to highlight the links between toxic chemicals and reproductive difficulties—that scientific studies support often resulting in miscarriage, infertility and congenital birth defects. This is a qualitative study that seeks to understand how women living close to hydraulic fracturing operations experienced reproductive difficulties, and how they gave meaning to these experiences.

DeLorme et al., in their article “*Communicating and Understanding Ecosystem Services Assessment With Coastal Stakeholders: Obstacles and Opportunities*,” report on insights and lessons learned from stakeholder engagement, particularly focus groups, conducted during a multi-year, National Oceanic Atmospheric Administration sponsored transdisciplinary project that sought to understand the benefits of natural and nature-based features in the northern Gulf of Mexico region, through the lens of economic impacts and ecosystem services. The results show that economic impacts and ecosystem services can be challenging to communicate due to the complexity of conceptualizing and evaluating. “The paper concludes with a discussion of future research opportunities for improving Ecosystem Services Assessment oriented science and outreach.”

Neenan et al., in the article “*Time to Listen: Children's Voice in Geoscience Education Research*,” highlight the importance of including children and young people, the future adults, in research and actions related to the social, political and educational dimensions of geoscience, as a way of focusing and including their voices in decisions and in the readiness to face climate change effects. It is the school-age generation that will be confronted with the worst effects of climate change, such as the greater frequency and intensity of extreme weather events, scarcity of water and food, increasing pollution and toxicity in human environments and in the human food chain, as well as higher order health crises. The present generation of children and youth is growing up in the context of the need to act now and to make difficult socio-political choices, inescapable at local and national levels (in order to) to manage resources and mitigate environmental damage. This article proposes the use of Children's Research Advisory Groups (CRAGs) to meaningfully include children and youth as co-researchers in geoscience-related research.

Fierros-González and López-Feldman in “*Farmers' Perception of Climate Change: A Review of the Literature for Latin America*,” present a review of original research articles published between 2000 and 2020, with the objective of highlighting the status of knowledge about farmers' perceptions and practices on adaptation to climate change in Latin America, highly vulnerable to climate change, also identify research gaps and inform future research. The authors point out, based on the revision done, that the available research is scarce (and), has been based mostly on qualitative analyses of case studies for a few countries. More research that identifies causal relationships is necessary. Data from surveys representative at national or subnational levels, as well as longitudinal data, will be very helpful to better understand farmers' perceptions. Finally, the use of field experiments and choice experiments can complement the use of observational data.

## Author contributions

All authors listed have made a substantial, direct, and intellectual contribution to the work and approved it for publication.

## Funding

This work was carried out at the R&D Unit Center for Functional Ecology - Science for People and the Planet (CFE), with reference UIDB/04004/2020, financed by FCT/MCTES through national funds (PIDDAC) Portugal, and also at the Institute of Social Sciences of the University of Lisbon (ICS-ULisboa), Portugal. This Research Topic was developed with the support of the European project-PHOENIX: The rise of citizen's voices for a Greener Europe- (contract ID: 101037328) funded by the European Commission under the EGD-European research priority Green Deal of the H2020 Program (H2020-EU.3.6).

## Conflict of interest

The authors declare that the research was conducted in the absence of any commercial or financial relationships that could be construed as a potential conflict of interest.

## Publisher's note

All claims expressed in this article are solely those of the authors and do not necessarily represent those of their affiliated organizations, or those of the publisher, the editors and the reviewers. Any product that may be evaluated in this article, or claim that may be made by its manufacturer, is not guaranteed or endorsed by the publisher.

